# Surgical timing for asphyxiating thoracic dystrophy

**DOI:** 10.1093/icvts/ivae141

**Published:** 2024-07-29

**Authors:** Xingfei Chen, Huilan Ye, Run Dang, Yiyu Yang

**Affiliations:** School of Pediatrics, Guangzhou Medical University, Guangzhou, China; School of Pediatrics, Guangzhou Medical University, Guangzhou, China; Pediatric Intensive Care Unit, Guangzhou Women and Children’s Medical Center, Guangzhou Medical University, Guangzhou, China; Pediatric Intensive Care Unit, Guangzhou Women and Children’s Medical Center, Guangzhou Medical University, Guangzhou, China

**Keywords:** Asphyxiating thoracic dystrophy, Thoracic deformity corrective surgery, Weaning from mechanical ventilation, Surgical timing

## Abstract

This report describes a 4-year-old girl diagnosed with asphyxiating thoracic dystrophy who experienced severe respiratory distress and multiple complications after undergoing a corrective operation for a thoracic deformity. The optimal age for children with asphyxiating thoracic dystrophy to receive a corrective operation is between 6 and 12 years old. For children under 6 years old, the decision to undergo an operation should be carefully evaluated.

## CASE REPORT

**Figure 1:**
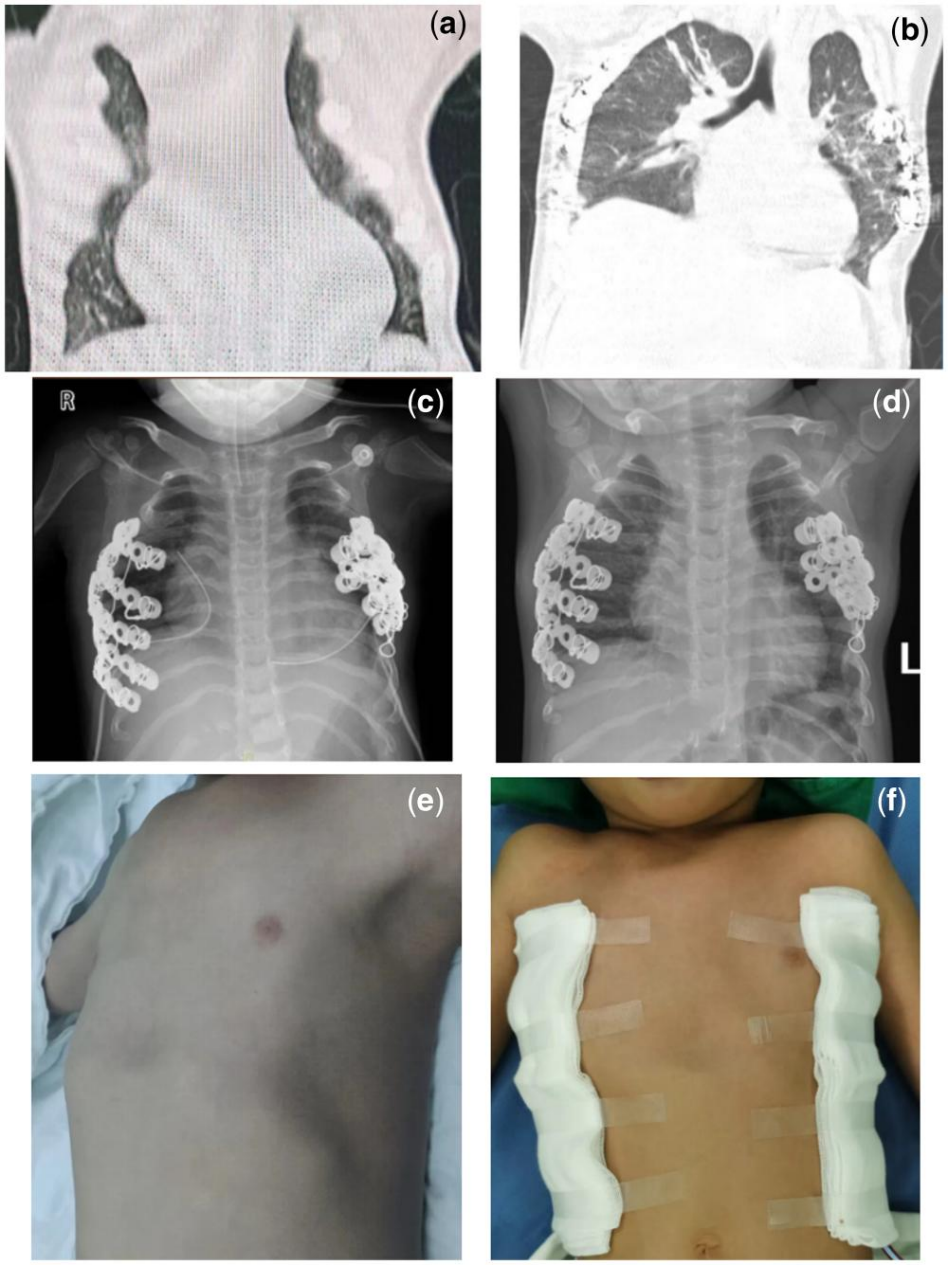
(**a**)Preoperative and (**b**)postoperative radiologic diagnosis using computed tomography; Radiologic diagnosis using digital radiology. (**c**) Four days postoperatively:1.Bilateral ribs show internal fixation with visible metal density. 2. light exudation in the lower left lung, suggestive of an inflammatory process. 3. The heart is mildly enlarged. (**d**) 93 days postoperatively: The lesions in both lungs were absorbed compared to before the operation; preoperative (**e**) and postoperative (**f**) thoracic photos.

A 4-year-old female presented with a progressive thoracic wall deformity existent since birth, accompanied by growth retardation and tachypnoea after exercise, with recurrent lung infections over the past 6 months. On 30 August 2023, she was diagnosed with asphyxiating thoracic dystrophy and underwent a corrective operation, which involved resection of 4 left and 5 right ribs, followed by the application of steel plates using Wang’s technique (Fig. 1a, e, and f). Although the operation was successful (Fig. 1b), the patient developed recurrent fever, tachycardia, worsening respiratory distress and decreased oxygen saturation. She was eventually transferred to the paediatric intensive care unit (Fig. 1c).

In the paediatric intensive care unit, she received mechanical ventilation and antimicrobial therapy. She subsequently developed gastrointestinal bleeding and was treated with fasting, fluid resuscitation and transfusions. However, she was observed to have poor limb muscle strength. Normal results were obtained from lumbar puncture, cranial computed tomography and genetic metabolic disease screening. After correcting her hypophosphataemia and discontinuing sedatives and analgesics, her muscle strength improved.

On postoperative day 48, she was weaned from the ventilator and transitioned to a high-flow nasal cannula. Although her oxygen saturation was maintained at a sufficient level, she experienced tachypnoea and an increased heart rate. Ten days after extubation, she developed a cough, and her heart rate and oxygen levels significantly decreased, requiring reintubation. On postoperative day 78, she was successfully weaned from the ventilator again and continued to use the high-flow nasal cannula. After 88 days of high-flow oxygen therapy, she was successfully transferred to a regular ward. She was discharged on postoperative day 93, without the need for oxygen support (Fig. 1d). Six months after discharge, she was hospitalized once for pneumonia, without the need for respiratory support, and was discharged 1 week later. Although her daily living abilities largely recovered, her physical activity had not yet returned to preoperative levels.

## DISCUSSION

The patient in this case report was diagnosed with asphyxiating thoracic dystrophy, a rare genetic bone disorder characterized by an abnormally narrow chest cavity, resulting in breathing difficulties and limited lung function. Despite normal intellectual development, the patient’s severe chest deformities, delayed physical development and recurrent respiratory infections required surgical intervention. Postoperatively, the patient experienced severe respiratory distress, infection, multiple-organ dysfunction and gastrointestinal bleeding, necessitating 3 months of respiratory support. These complications highlight the importance of assessing surgical risks. We investigated the causes of prolonged weaning difficulty in patients and analyzed the optimal timing for surgery in patients with these chest deformities.

Most studies suggest that within a certain age range, younger patients experience fewer complications and better clinical outcomes. According to an investigation based on expert opinions, the Chinese Association of Thoracic Surgeons recommends that the optimal age for surgical treatment of funnel chest is between 6 and 12 years old [1]. Some studies suggest that school-aged children (6–12 years old) experience significantly fewer surgical complications, less blood loss and shorter periods of hospitalization compared to older patients (>12 years old) [2]. Another study found that the improvement in cardiopulmonary function after the operation was better in patients aged 3–12 years old than in those over 12 years old, which supported early intervention [3]. Moreover, earlier corrective surgery may be more beneficial for the social and psychological development of children with thoracic deformities. However, at the Children’s Hospital of The King’s Daughters, surgeons prefer to perform minimally invasive repair of pectus excavatum on patients aged 12 to15 years, because the chest wall remains malleable, ensuring quick recovery and low recurrence rates [4].

The patient’s difficulty in weaning from the ventilator may be attributed to 3 main factors: (i) the patient was young (4 years old), had a low body weight (9 kg) and poor nutritional status, which reduced surgical tolerance. (ii) The surgical removal of 9 ribs resulted in significant postoperative pain, and the implanted steel plate limited chest expansion, leading to restrictive ventilation disorders, which may be difficult for a child’s respiratory muscles to compensate for in the short term. (iii) The long duration of the operation increased the risk of postoperative infection.

In recent years, study results have suggested that tracheotomy may reduce respiratory resistance and aid children’s autonomous breathing. However, due to the child’s anatomical structure, incomplete development, high risk, multiple postoperative complications and low parental acceptance, we did not implement a tracheotomy. Reflecting on the weaning difficulty in this case, tracheotomy may be helpful for patients with mechanical ventilation challenges.

## CONCLUSION

Based on the literature and the challenges encountered in this case, corrective surgery for thoracic deformities in children under 6 years old necessitates greater caution. Surgeons should conduct thorough preoperative assessments of the child’s age, weight, overall health and surgical tolerance to mitigate surgical trauma, shorten operative time and reduce the risks of postoperative infections and complications.

## ETHICS STATEMENT

Informed consent forms have been signed.

Medical Ethics Committee of Guangzhou Women and Children’s Medical Center

穗妇儿 科伦 批字

[2024]第 215A01 号。: [2024] No. 215A01

## Data Availability

All relevant data are within the manuscript and its Supporting Information files.
